# Mechanisims of asthma and allergic disease – 1090. Eosinophils enhance airway smooth muscle cell proliferation via the release of cystl leukotrines

**DOI:** 10.1186/1939-4551-6-S1-P86

**Published:** 2013-04-23

**Authors:** Saleh Al Muhsen, Rabih Halwani, Hamdan Al Jahdali, Qutayba Hamid

**Affiliations:** 1 Prince Naif Center for Immunology Research, College of Medicine, King Saud University, Riyadh, Saudi Arabia; 2 King Saud University for Health Sciences, Internal Medicine, Riyadh, Saudi Arabia; 3 Meakins-Christie Laboratories, Mcgill University, Montreal, Canada

## Background

Asthma is a chronic inflammatory disorder of the lung airways that is associated with airway remodeling and hyperresponsiveness. Its is well documented that the smooth muscle mass in asthmatic airways is increased due to hypertrophy and hyperplasia of the ASM cells. Moreover, eosinophils have been proposed in different studies to play a major role in airway remodeling. Here, we hypothesized that eosinophils modulate the airways through enhancing ASM cell proliferation. The aim of this study is to examine the effect of eosinophils on ASM cell proliferation using eosinophils isolated from asthmatic and normal.

## Methods

Eosinophils were isolated from peripheral blood of 6 mild asthmatics and 6 normal control subjects. ASM cells were incubated with eosinophils or eosinophil membranes and ASM proliferation was estimated using thymidine incorporation. The mRNA expression of extracellular matrix (ECM) in ASM cells was measured using quantitative real-time PCR. The effect of eosinophil-derived proliferative cytokines on ASM cells was determined using neutralizing antibodies. The role of eosinophil derived Cysteinyl Leukotrienes in enhancing ASM was also investigated.

## Results

Co-culture with eosinophils significantly increased ASM cell proliferation. However, there was no significant difference in ASM proliferation following incubation with eosinophils from asthmatic versus normal control subjects. Co-culture with eosinophil membranes had no effect on ASM proliferation. Moreover, there was no significant change in the mRNA expression of ECM proteins in ASM cells following co-culture with eosinophils when compared with medium alone. Interestingly, blocking the activity of cysteinyl Leukotries using antagonists inhibited eosinophil-derived ASM proliferation.

## Conclusions

Eosinophils enhances the proliferation of ASM cells. This role of eosinophil does not seem to depend on ASM derived ECM proteins nor on Eosinophil derived TGF-β or TNF-α. Eosinophil seems to induce ASM proliferation via the secretion of Cysteinyl Leukotrienes.

